# Editorial: New Developments With Magnetoelectrical Techniques in Schizophrenia

**DOI:** 10.3389/fpsyt.2021.733033

**Published:** 2021-08-11

**Authors:** Vicente Molina, Alvaro Díez-Revuelta, Cristopher Mulert

**Affiliations:** ^1^Department of Psychiatry, School of Medicine, University of Valladolid, Valladolid, Spain; ^2^Psychiatry Service, University Hospital of Valladolid, Valladolid, Spain; ^3^Department of Psychiatry, University of Giessen, Giessen, Germany

**Keywords:** schizophrenia, electroencephalography, magnetoencepalography, GABA, glutamate

The magnetoelectrical techniques have a long history in Psychiatry since its early discovery by Hans Berger. Although neuroimaging, in particular magnetic resonance, has become the standard approach to assess structural, functional, and even biochemical alterations in the living patient, electroencephalography (EEG) and magnetoencephalography (MEG) still have a role in research in mental illness. The reason for this is quite straightforward: with these techniques, researchers can assess the biological correlates nearest to the functions altered in psychiatric patients. In other words, mental functions are based on the ever-changing depolarization and repolarization of neural groups or assemblies, whose direct and measurable consequence is the bioelectrical activity detected by EEG and MEG. The local and global patterns of such bioelectrical dynamics change in miliseconds, a temporal resolution affordable for MEG and EEG but not for other functional techniques.

Older qualitative assessments of EEG, based on the visual inspection of the EEG or even on amplitude and latency of event-related potential have yielded some important fruits for research in schizophrenia. As an example, the reduced amplitude of the P300 event-related potential in this syndrome. However, recent advances have largely broadened the possibilities offered by these techniques. It is now possible, for example, to combine EEG and MEG measurements of connectivity based on phase-locking of the signals recorded in each sensor with graph-theory. This may help describing the global pattern of brain activity in relation to relevant properties of the cerebral network, such as its degree of specialization/segregation, or the equilibrium between specialization and integration known as the small-world-index. In this number, Hamilton and Northoff review the literature on the biolelectrical basis of self-disturbances in schizophrenia assessed with different study paradigms. These include odd-ball tasks (related to sensitivity to aberrant salience according to the authors) and self-referential processing and source monitoring aimed at eliciting corollary discharge (i.e., the signal sent by regions initiating a motor act to the corresponding sensory areas that allow identifying such act as our own), among others. The authors conclude that N100 suppression in source monitoring is a robust finding in schizophrenia, likely associated to a weaker corollary discharge. However, clinical instruments across the studies included in this review do not allow a direct assessment of alterations in self-experience, which remains to be done. Source monitoring probably allow for the most direct assessment of self-experience among the available paradigms. However, in previous reports differences in these tasks in schizophrenia have been only studied in association to positive and negative symptoms (Hamilton and Northoff), only bearing a partial relation to self-disturbances.

A relatively new development of magnetoelectrical techniques makes it feasible to apply it directly for therapeutic purposes. Trascranial magnetic stimulation (TMS) has shown a relevant effect in the treatment of depression. In schizophrenia, research paradigms such as long interval cortical inhibition (LICI), short interval cortical inhibition SICI and intra-cortical facilitation (ICF) allow for a direct, *in vivo* assessment of the inhibitory/excitatory equilibrium, which has a relevant potential in schizophrenia research (1). Theta burst stimulation (TBS) is a novel approach to the treatment of depression with TMS. In this issue, Wu et al. report on the use of intermittent TBS for schizophrenia. Although the sample is small, the results suggest the possibility of improving cognitive function in schizophrenia, an area where significant contributions are much needed.

Magnetoelectrical techniques may contribute to the study of the inhibitory/excitatory equilibrium in other interesting ways, such as the assessment of the bioelectrical effects of drugs affecting it in reasonably known ways. In the present issue Curic et al. assess the effects on EEG of administering ketamine to healthy volunteers. In them, resting state connectivity and source analyses were carried out, as well as exams of psychotic symptoms and altered states of consciousness associated to ketamine. The authors found an increase in resting oscillatory power in theta and gamma band, located in middle regions for theta and widespread (except for frontal) for gamma bands. Changes in the gamma band were associated to negative symptoms scores. As the authors discuss, these bioelectrical effects may be coherent with the consequences of ketamine administration on the inhibitory system, whose function may result decreased as a consequence of decreased stimulation of NMDA receptors on GABA cells by ketamine (2). Remarkably, ketamine is known to induce both cognitive, positive and negative symptoms similar to those of schizophrenia, and elevated power in the theta and gamma band have been also reported in this syndrome. In this context, the study of Curic et al. support the role of a cortical hyperactivation in schizophrenia that may hamper adequate context-related modulation in a similar was as what has been reported using functional magnetic resonance (3).

One important handicap for the use of EEG in psychiatric assessments, both clinical and basic, is the potentially powerful effects of treatment with antipsychotic and other psychopharmacological drugs on bioelectrical signals. These effects are difficult to rule out with usual approaches, such as temporary interruptions of treatments because of ethical reasons and the likely resulting protracted neuronal changes, especially when patients have received such treatments for a long period. Therefore, studies of EEG in cases in the early stages of schizophrenia and risk states may help elucidating the alterations properly related to illness and those to treatments. In the present issue, Perrottelli et al. show a relevant systematic review in patients in the early or prodromal stages of the schizophrenia spectrum. This review reveals that multiple EEG indices were already altered in at risk-mental states and early stages of schizophrenia in the same direction as in later stages. For instance, higher resting power in the delta and gamma power (the results were more controversial for the theta band) and lower amplitudes in event related potentials associated to sensory gating (P50, N100) and context updating (P300). Interestingly, the P3b potential decreased in at-risk subjects transiting to psychosis. Although a direct effect of treatment was not assessed in this review, the data revealed that at least some of the EEG alterations found in chronic schizophrenia (including resting state hyperactivity and reduced evoked activity) may not be an effect of confounders. Therefore, at least some of the EEG alterations may be characteristic of this syndrome, or at least of some biotypes within int.

## Author Contributions

All authors listed have made a substantial, direct and intellectual contribution to the work, and approved it for publication.

## Conflict of Interest

The authors declare that the research was conducted in the absence of any commercial or financial relationships that could be construed as a potential conflict of interest.

## Publisher's Note

All claims expressed in this article are solely those of the authors and do not necessarily represent those of their affiliated organizations, or those of the publisher, the editors and the reviewers. Any product that may be evaluated in this article, or claim that may be made by its manufacturer, is not guaranteed or endorsed by the publisher.
